# The complexity of glucose time series is associated with short- and long-term mortality in critically ill adults: a multi-center, prospective, observational study

**DOI:** 10.1007/s40618-024-02393-4

**Published:** 2024-05-18

**Authors:** Y. Wang, S. Li, J. Lu, K. Feng, X. Huang, F. Hu, M. Sun, Y. Zou, Y. Li, W. Huang, J. Zhou

**Affiliations:** 1 Department of Endocrinology and Metabolism, Shanghai Sixth People’s Hospital Affiliated to Shanghai Jiao Tong University School of Medicine; Shanghai Clinical Center for Diabetes; Shanghai Diabetes Institute; Shanghai Key Laboratory of Diabetes Mellitus, 600 Yishan Road, Shanghai, 200233 China; 2 Department of Anesthesiology, Tongji University Affiliated Shanghai Tenth People’s Hospital, Shanghai, China; 3 Department of Critical Care Medicine, Shanghai Sixth People’s Hospital Affiliated to Shanghai Jiao Tong University School of Medicine, 600 Yishan Road, Shanghai, 200233 China; 4 Department of Critical Care Medicine, Jinshan Branch of Shanghai Sixth People’s Hospital, Shanghai, China; 5 Department of Critical Care Medicine, Shanghai Fengxian District Central Hospital, Shanghai, China; 6 Department of Critical Care Medicine, Shanghai Eighth People’s Hospital, Shanghai, China; 7 Department of Critical Care Medicine, Shanghai Sixth People’s Hospital East Campus, Shanghai, China; 8 Department of Critical Care Medicine, Tongji University Affiliated Shanghai Tenth People’s Hospital, 301 Yanan Middle Road, Shanghai, 200040 China; 9 Department of Critical Care Medicine, Shanghai Xuhui Central Hospital, Zhongshan-Xuhui Hospital, Fudan University, 966 Huaihai Middle Road, Shanghai, 200031 China

**Keywords:** Complexity of glucose time series, Continuous glucose monitoring, Mortality, Critically ill patients

## Abstract

**Background:**

The wealth of data taken from continuous glucose monitoring (CGM) remains to be fully used. We aimed to evaluate the relationship between a promising new CGM metric, complexity of glucose time series index (CGI), and mortality in critically ill patients.

**Methods:**

A total of 293 patients admitted to mixed medical/surgical intensive care units from 5 medical centers in Shanghai were prospectively included between May 2020 and November 2021. CGI was assessed using intermittently scanned CGM, with a median monitoring period of 12.0 days. Outcome measures included short- and long-term mortality.

**Results:**

During a median follow-up period of 1.7 years, a total of 139 (47.4%) deaths were identified, of which 73 (24.9%) occurred within the first 30 days after ICU admission, and 103 (35.2%) within 90 days. The multivariable-adjusted HRs for 30-day mortality across ascending tertiles of CGI were 1.00 (reference), 0.68 (95% CI 0.38–1.22) and 0.36 (95% CI 0.19–0.70), respectively. For per 1-SD increase in CGI, the risk of 30-day mortality was decreased by 51% (HR 0.49, 95% CI 0.35–0.69). Further adjustment for HbA1c, mean glucose during hospitalization and glucose variability partially attenuated these associations, although the link between CGI and 30-day mortality remained significant (per 1-SD increase: HR 0.57, 95% CI 0.40–0.83). Similar results were observed when 90-day mortality was considered as the outcome. Furthermore, CGI was also significantly and independently associated with long-term mortality (per 1-SD increase: HR 0.77, 95% CI 0.61–0.97).

**Conclusions:**

In critically ill patients, CGI is significantly associated with short- and long-term mortality.

**Supplementary Information:**

The online version contains supplementary material available at 10.1007/s40618-024-02393-4.

## Introduction

Over the past few years, the adoption of continuous glucose monitoring (CGM) has grown rapidly as a result of improvements in sensor accuracy, greater convenience and ease of use. Accumulating evidence suggest that the use of CGM has the potential to further improve glucose control, not only in real-life experience but also within the hospital [1–5]. Meanwhile, previous studies have also realed the potential of CGM as a better tool for glycemic assessment in various populations both with and without diabetes [6, 7]. However, it should be noted that currently most proposed CGM metrics simplify data by using linear calculation methods and only assess the amplitude changes of glucose, which may fail to reveal the internal structure of dynamical glucose regulation system [8]. The wealth of data taken from CGM remains to be fully used.

Time series analysis allows for the quantification of complex non-linear characteristics in continuously monitored physiological signals. This method has been extensively employed to study various biological phenomena, such as heart rate, electroencephalogram, and temperature, contributing to a better understanding of functional dysregulation [9–14], but received less attention in glucose monitoring. In our previous study [15], we found that the complexity of glucose time series index (CGI), derived from CGM data, decreased progressively from healthy controls to subjects with impaired glucose regulation and then to type 2 diabetes. Moreover, CGI was significantly associated with β-cell function even after adjusting for insulin sensitivity. More recently, we found that lower CGI is associated with an increased risk of all-cause mortality among type 2 diabetes achieving the glycated hemoglobin A1c (HbA1c) target [16]. These existing data indicate the potential value of CGI as a novel and sensitive marker for the status of glucose homeostasis. We hypothesized that low CGI may represent a failing glucose regulatory system, which is unable to correct glucose concentrations frequently and quickly. However, currently evidence linking CGI to outcomes is still very limited.

Furthermore, critically ill patients are at increased risk for dysglycemia. In contrast with the substantial evidence linking CGM metrics to adverse outcomes in the outpatient setting, there are scarce data regarding the hospital setting, particularly within the intensive care units. In this context, based on a multi-center, prospective, observational cohort, the present study aimed to examine the relationship between CGI derived from 14-day isCGM data and mortality risk in critically ill patients.

## Methods

### Study design and population

We used data from the INDices of contInuous Glucose monitoring and adverse Outcomes in Intensive Care Units (INDIGO-ICU) study. The rationale and methodology have been described previously [17]. Briefly, this study aimed to longitudinally examine the effects of quality of glucose control assessed by CGM on mortality risk in critically ill patients. It was designed as a multi-center, prospective, and observational cohort study. The INDIGO-ICU study was approved by the Research Ethics Committees of Shanghai Sixth People's Hospital Affiliated to Shanghai Jiao Tong University School of Medicine. The study was in accordance with the Helsinki Declaration principles, and informed consent was obtained from all participant.

Patients admitted to mixed medical/surgical ICUs of 5 medical centers in Shanghai were consecutively recruited between May 2020 and November 2021. Patients were included if they were adults (age ≥ 18 years) and expected to stay in intensive care units for at least 3 days. Exclusion criteria involved: (1) readmission to the ICU; (2) fewer than 24-h CGM data; (3) receiving high-dose ascorbic acid or acetaminophen > 4 g/day [18–20]; (4) an admitting diagnosis of diabetic ketoacidosis or hyperosmolar hyperglycemic state. Finally, a total of 293 participants was included in the analysis (Supplementary Fig. 1).

### Glucose control strategy

The standard ICU’s glucose control protocol has been described previously [17]. In brief, the blood glucose target was 140–180 mg/dL (7.8–10.0 mmol/L) in our study. The blood glucose testings were performed using venous or capillary blood. The frequency of blood glucose monitoring ranged from hourly to every 4 to 6 h based on clinical need. Continuous intravenous (IV) regular insulin infusion was initiated when blood glucose exceeded 180 mg/dL on 2 successive readings. For milder degrees of hyperglycemia, subcutaneous short acting analogue insulin was administered.

### Assessment of CGI

The methodology of CGM (FreeStyle Libre CGM Pro; Abbott Diabetes Care, Alameda, CA) has been described previously [17]. In brief, the sensors were inserted on the first day of ICU admission. Then interstitial glucose levels were continuously measured every 15 min, generating a daily record of 96 glucose values for up to 14 days. Overall, patients wore the sensor for a median (intequartile range [IQR]) period of 12.0 [7.0–14.0] days during their hospitalization. The patients and the treating physicians were blinded to the CGM glucose results during the research.

Refined composite multi-scale entropy (RCMSE) analysis were performed to calculate CGI, as previously described [15, 16]. Details about the calculation method have been provided in supplementary material. In brief, first, the raw glucose data during a 24-h period was divided into several equally distributed and non-overlapping time windows according to predefined time scales, a process called coarse-graining. The time scales in this study were set at 1–4, which corresponded to the intervals of 15–60 min, as the CGM system recorded glucose readings every 15 min. For each coarse-grained time series, data points inside segmented windows were averaged to form a new set of time series data. Next, the entropy of each coarse-grained time series was calculated according to the RCMSE algorithm. For each participant, four entropy values corresponded to time scale of 1–4 were generated. Finally, CGI was calculated as the sum of the four entropy values generated in previous step. We averaged the CGI values obtained for each day, resulting in one mean value per patient. Higher CGI reflects stronger unpredictability of the glucose dynamics. Meanwhile, mean glucose and glucose coefficient of variation (CV) were also calculated using CGM data [21, 22].

### Data extraction, prospective follow-up and outcome

The methodology of data extraction has been described previously [17]. In brief, clinically relevant data was extracted from the hospital electronic medical record system and ICU’s comprehensive database, including demographic information, anthropometric measures and laboratory results, which were recorded within the first 24 h of ICU admission. Diabetes status was assigned at the time of ICU admission for all patients based on all available information including medical history and electronic databases of outpatient medication administration.

Information on death after discharge from hospitalization was additionally obtained from the database of the Shanghai Municipal Center for Disease Control and Prevention in the current study, which was linked by personal identification number. We used chart review to evaluate the confirmation of death (COD) via the Shanghai adaptation of the Medical Record Audit Form. The evaluation of COD was conducted as described in detail previously [16, 23]. All participants were followed up until a death event occurred or until 7 March 2023, whichever occurred first. Accordingly, outcome measures were defined as short-term all-cause mortality (30-day mortality and 90-day mortality) and long-term all-cause mortality (mortality at the end of follow-up) in the current study.

### Statistical analysis

R version 4.0.3 was used for the statistical analysis. Continuous variables and categorical variables were presented as mean ± standard deviation (SD) and *n* (%), respectively. To test the trends of different risk factors across tertiles of CGI, ANOVA tests or Jonckheere–Terpstra tests were conducted for normally or non–normally distributed continuous variables, and Cochran‐Armitage tests for categorical variables.

Potential nonlinear associations between the levels of CGI and mortality were examined using restricted cubic spline analysis. Kaplan–Meier survival curves were plotted and analyzed by log-rank tests. Cox proportional hazards regression was performed to assess the relationships of CGI, as either categorical (tertiles: ≤ 2.10 [ref.], 2.11–2.74, > 2.74) or continuous variable (per 1-SD increase), with the risk of mortality. The analyses were first carried out without adjustment and then adjusted for covariates, which were selected based on prior literature and the univariable analysis results. Three models were used: the first model (main model) was adjusted for sex, age, APACHE II score, mechanical ventilation, creatinine, diabetes, use of glucocorticoid and use of insulin in the hospital; the second model was additionally adjusted for HbA1c levels; and the third model was additionally adjusted for mean glucose and CV derived from CGM during hospitalization. Two-tailed *P* values < 0.05 were considered to indicate statistical significance.

## Results

A total of 293 critically ill patients were included in the final analysis. The clinical characteristics of these participants have been previously reported [17]. In brief, at baseline, the mean age was 68 ± 15 years, 67.6% were males, and the mean APACHE II score was 19 ± 6. Diabetes was present in 23.5% of the participants. The clinical characteristics of the study population across CGI tertiles are presented in Table 1. Briefly, participants with higher CGI were younger, had lower proportions of diabetes and had lower levels of HbA1c, mean glucose and CV (all P for trend < 0.05).

**Table 1:** Characteristics of participants by tertiles of CGI

	CGI			*P* for trend
	Tertile 1 (*n* = 99)	Tertile 2 (*n* = 96)	Tertile 3 (*n* = 98)	
*Patient characteristics*				
Male, *n* (%)	71 (71.7)	62 (64.6)	65 (66.3)	0.419
Age, years	72 ± 14	66 ± 15	68 ± 16	0.023
Diabetes, *n* (%)	38 (38.4)	22 (22.9)	9 (9.2)	< 0.001
APACHE II score	20 ± 6	19 ± 6	19 ± 6	0.607
Mechanical ventilation, *n* (%)	87 (87.9)	83 (86.5)	79 (80.6)	0.320
Systolic blood pressure, mmHg	129 ± 27	135 ± 26	138 ± 30	0.121
Diastolic blood pressure, mmHg	76 ± 14	74 ± 18	76 ± 19	0.982
Heart rate, beats/min	91 ± 19	91 ± 18	92 ± 22	0.702
Respiratory rate, breaths/min	20 ± 6	19 ± 4	23 ± 13	0.662
Use of glucocorticoid in hospital, *n* (%)	24 (24.2)	18 (18.8)	11 (11.2)	0.018
Use of insulin in hospital, *n* (%)	38 (38.4)	27 (28.1)	6 (6.1)	< 0.001
Doses of insulin, U/day^†^	39 ± 21	36 ± 20	31 ± 10	0.689
*Laboratory results*				
HbA1c, %	7.0 ± 1.9	6.3 ± 1.8	5.7 ± 1.0	< 0.001
WBC, × 10^9^/L	12.5 ± 7.1	13.3 ± 6.3	11.8 ± 5.8	0.766
Hemoglobin, g/L	109 ± 42	106 ± 39	100 ± 23	0.103
Creatinine, μmol/L	142 ± 149	113 ± 103	145 ± 187	0.052
pH	7.4 ± 0.1	7.4 ± 0.3	7.4 ± 0.1	0.200
Na^+^, mmol/L	141 ± 10	139 ± 7	138 ± 7	0.578
HCT, %	33 ± 8	32 ± 8	32 ± 9	0.138
*CGM metrics*				
Mean glucose, mmol/L	9.8 ± 2.9	7.5 ± 2.6	6.3 ± 1.8	< 0.001
Coefficient of variation, %	30.3 ± 7.5	29.3 ± 7.1	24.5 ± 6.4	< 0.001

Data are expressed as mean ± standard deviation or *n* (%)

*CGI*, complexity of glucose time series index; *APACHE II*, Acute Physiology Score of the Acute Physiology and Chronic Health Evaluation II; *HbA1c*, glycated hemoglobin A1c; *WBC*, white Blood Cell; *HCT*, hematocrit; *CGM*, continuous glucose monitoring

^†^Doses of insulin were reported only for patients who have used insulin in the hospital (*n* = 71)

As shown in Supplementary Table 1, CGI showed a weak correlation with HbA1c (*r* = − 0.28), and moderate correlations with hyperglycemia, hypoglycemia and glycemic variability metrics derived from CGM during hospitalization (|*r*|= 0.34–0.60), including mean sensor glucose, time above range > 180 mg/dl (10 mmol/L), time in range 70–180 mg/dl (3.9–10 mmol/L), time below range < 70 mg/dl (3.9 mmol/L), CV and standard deviation (SD).

### Association between CGI and short-term mortality

**Fig. 1:**
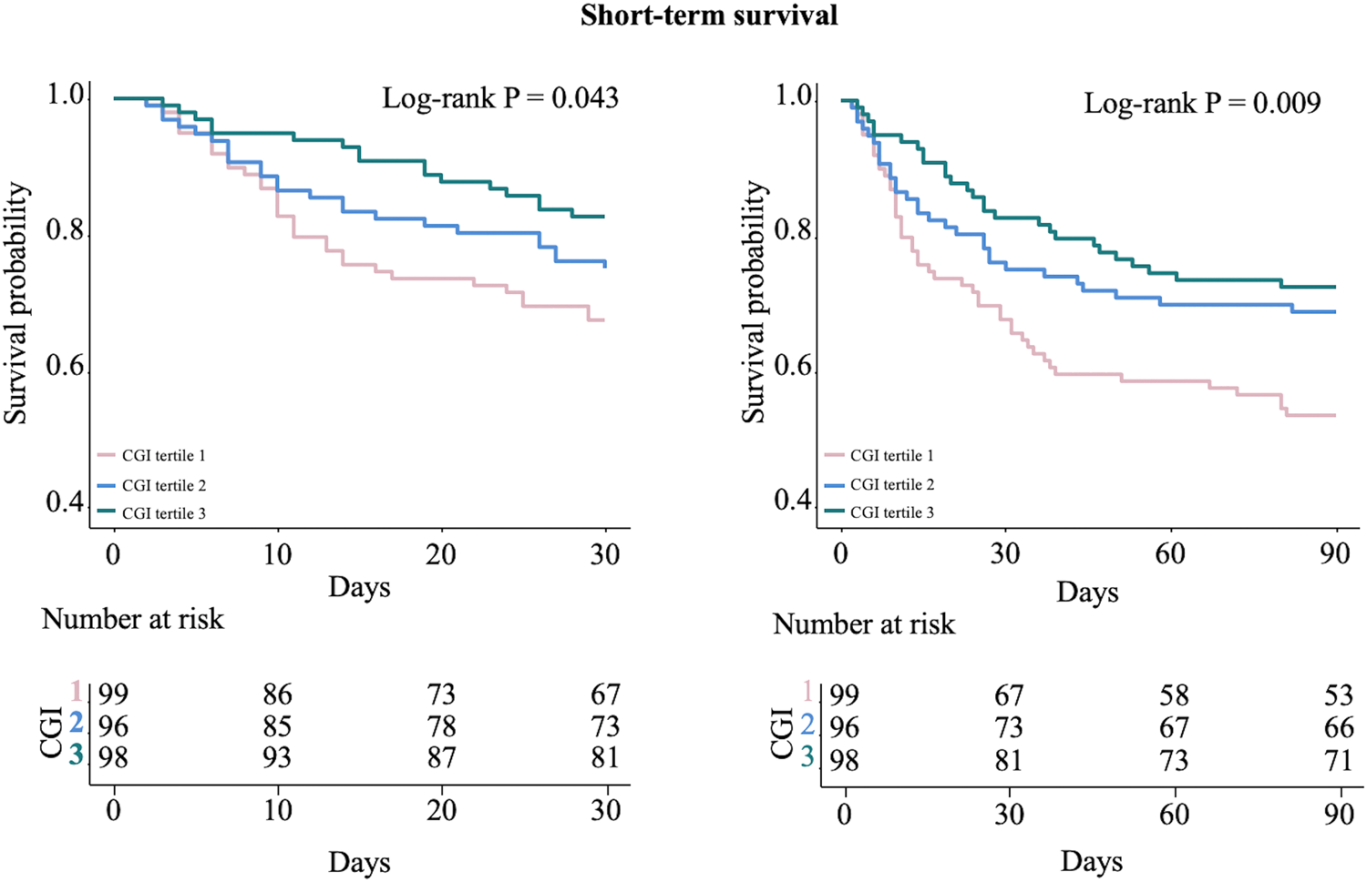
Kaplan–Meier curves for short-term survival including 30-day survival and 90-day survival according to CGI tertiles in critically ill patients. *CGI*, complexity of glucose time series index

73 (24.9%) deaths were identified within the first 30 days after ICU admission, and 103 (35.2%) within 90 days. The Kaplan–Meier curves showed that both the 30-day survival probability (log-rank P = 0.043) and the 90-day survival probability (log-rank P = 0.009) were gradually improved as CGI tertiles ascended (Fig. 1). Multivariable-adjusted restricted cubic spline analysis suggested a significant negative linear association between CGI and short-term mortality (both P for nonlinearity > 0.05, Supplementary Fig. 2A). The multivariable-adjusted (sex, age, APACHE II score, mechanical ventilation, creatinine, diabetes, use of glucocorticoid, and use of insulin) hazard ratios (HRs) for 30-day mortality across ascending tertiles of CGI were 1.00 (reference), 0.68 (95% CI 0.38–1.22) and 0.36 (95% CI 0.19–0.70), respectively. For per 1-SD increase in CGI, the risk of 30-day mortality was decreased by 51% (HR 0.49, 95% CI 0.35–0.69) (Table 2). Further adjustment for HbA1c, mean glucose during hospitalization and CV partially attenuated these associations, although the link between CGI and 30-day mortality remained significant (per 1-SD increase: HR 0.57, 95% CI 0.40–0.83, Fig. 3). Similar results were observed when 90-day mortality was considered as the outcome (Table 2, Fig. 3).

**Table 2:** Hazard ratios of CGI for short- and long-term mortality in critically ill patients

	CGI			Per 1-SD increase
	Tertile 1 (≤ 2.10)	Tertile 2 (2.11 ~ 2.74)	Tertile 3 (> 2.74)	
*Short-term mortality*				
30-day mortality				
No. of participants	99	96	98	–
No. of cases	32	24	17	–
Crude	1.00	0.73 (0.43–1.24)	0.48 (0.27–0.86)	0.59 (0.44–0.79)
Main model	1.00	0.68 (0.38–1.22)	0.36 (0.19–0.70)	0.49 (0.35–0.69)
90-day mortality				
No. of participants	99	96	98	–
No. of cases	46	30	27	–
Crude	1.00	0.61 (0.39–0.97)	0.51 (0.31–0.81)	0.60 (0.47–0.78)
Main model	1.00	0.68 (0.42–1.12)	0.47 (0.28–0.81)	0.58 (0.44–0.76)
Long-term mortality				
No. of participants	99	96	98	–
No. of cases	58	40	41	–
Crude	1.00	0.62 (0.42–0.93)	0.58 (0.39–0.87)	0.72 (0.59–0.87)
Main model	1.00	0.72 (0.47–1.11)	0.58 (0.37–0.91)	0.71 (0.57–0.88)

Main model was adjusted for sex, age, APACHE II score, mechanical ventilation, creatinine, diabetes, use of glucocorticoid in the hospital and use of insulin in the hospital

*CGI*, complexity of glucose time series index

### Association between CGI and long-term mortality

**Fig. 2:**
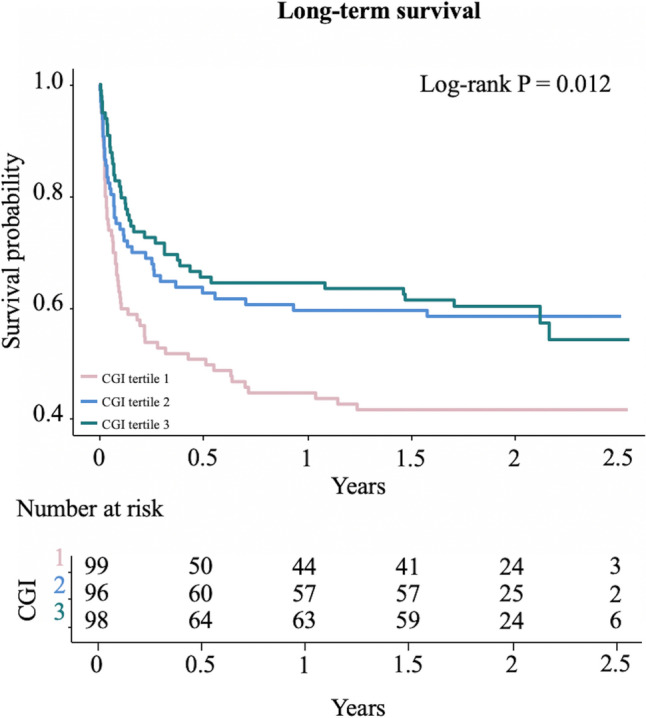
Kaplan–Meier curves for long-term survival according to CGI tertiles in critically ill patients. *CGI*, complexity of glucose time series index

**Fig. 3:**
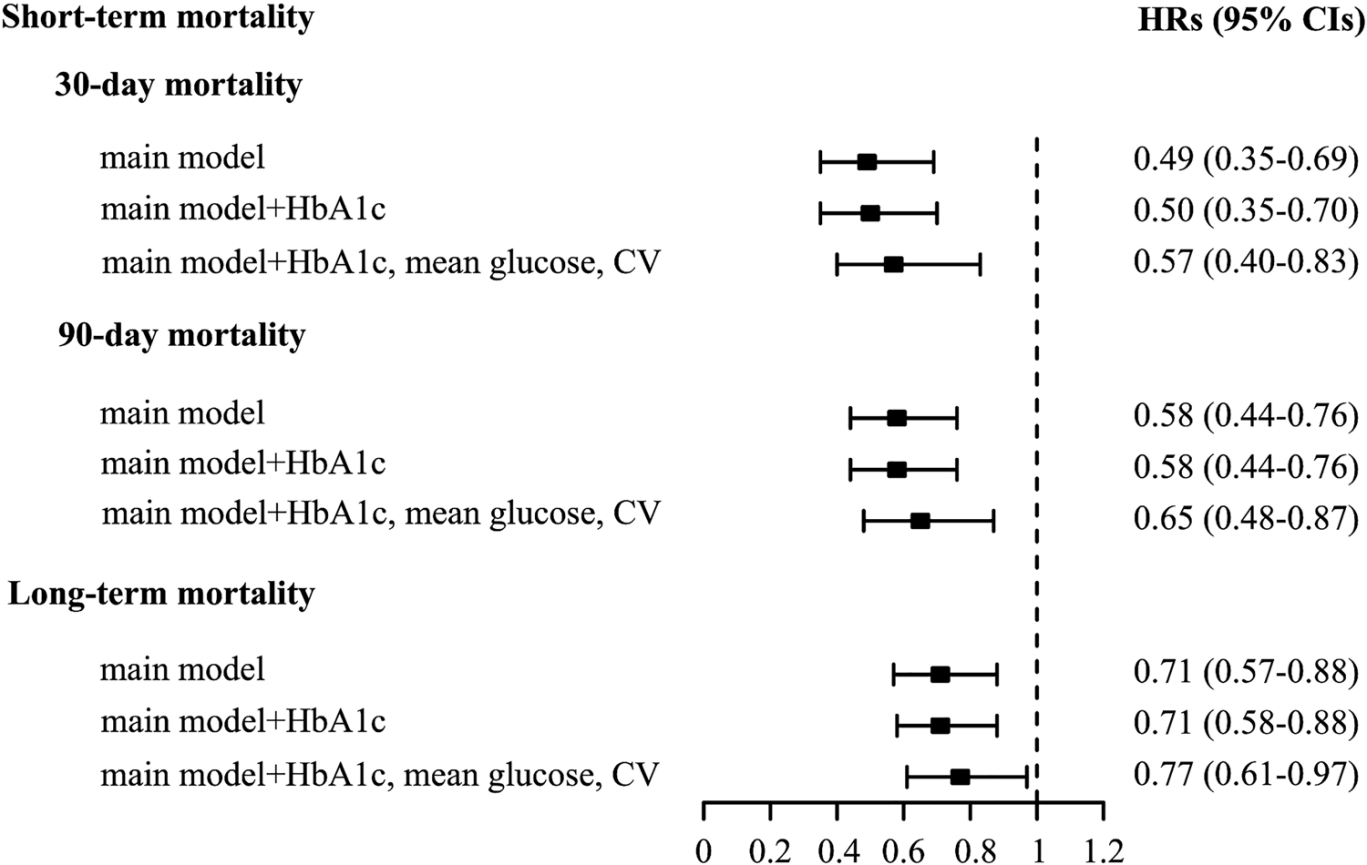
Hazard ratios of CGI (per 1-SD increase) for short- and long-term mortality in critically ill patients, following additional adjustment for HbA1c, mean glucose during hospitalization and CV to the main model. The main model was adjusted for sex, age, APACHE II score, mechanical ventilation, creatinine, diabetes, use of glucocorticoid in the hospital and use of insulin in the hospital. *CGI*, complexity of glucose time series index; *HbA1c*, glycated hemoglobin A1c; *CV*, glucose coefficient of variation

During a median follow-up period of 1.7 years, 139 participants (47.4%) died. The Kaplan–Meier curves showed that patients with the lowest tertile of CGI had worse overall survival rate, compared with those with the middle and highest tertiles of CGI (log-rank P = 0.012, Fig. 2). Multivariable-adjusted restricted cubic spline analysis also suggested a significant negative linear association between CGI and long-term mortality (P for nonlinearity > 0.05, Supplementary Fig. 2B). The multivariable-adjusted HRs for long-term mortality across ascending tertiles of CGI were 1.00 (reference), 0.72 (95% CI 0.47–1.11) and 0.58 (95% CI 0.37–0.91), respectively. For per 1-SD increase in CGI, the risk of long-term mortality was decreased by 29% (HR 0.71, 95% CI 0.57–0.88) (Table 2). After further adjustment for HbA1c, mean glucose during hospitalization and CV, CGI was marginally significantly associated with long-term mortality (per 1-SD increase: HR 0.77, 95% CI 0.61–0.97) (Fig. 3).

## Discussion

In this multi-center, prospective cohort of 293 critically ill patients, we observed a significant association between higher levels of CGI, assessed by 14-day CGM, and lower risk of both short- and long-term mortality. Even after further adjustment for HbA1c, mean glucose during hospitalization and CV, the association between CGI and mortality remained significant. Our data suggests that CGI holds promise to serve as a new marker for poor prognosis in critically ill patients.

The introduction of CGM technology has provided an opportunity for novel and in-depth insights into glucose regulation. As a new indicator derived from CGM data through time series analysis, glucose complexity may provide complementary—and perhaps more powerful—information on glucose regulation than conventional glycemic analysis. [15, 24–30] However, currently evidence linking glucose complexity to adverse clinical outcomes is still very limited. For critically ill patients, in 2010, a pilot study involving 42 patients found that glucose complexity, evaluated by detrended fluctuation analysis, is associated with higher mortality [31]. Later in 2012, this association were further confirmed in a post-hoc analysis of 174 patients from two randomized controlled trials [32]. Unlike these two studies with either small sample size or retrospective design, our study was conducted in a multi-center, prospective cohort of 293 patients. In addition, a new CGM technology as well as a new calculation method for glucose complexity were used in the current study and patients were monitored for longer period, with a median of 12 days. Moreover, we considered long-term mortality as an outcome, and found a significant association between CGI and long-term mortality as well.

The underlying mechanisms under the close association between CGI and mortality in critically ill patients can only be speculated. A seductive explanation could be based on the the mild to moderate correlations of CGI with hyperglycemia, hypoglycemia and glycemic variability measures. In line with this, the predictive power of CGI was partially diminished following adjustments for HbA1c, mean glucose during hospitalization, and CV in our study. Therefore, on the one hand, CGI may offer a valuable view of abnormal glucose regulation reflected by these conventional glycemic metrics, which has been well established to be associated with mortality by excessive counterregulatory hormones, production of inflammatory cytokines and reactive oxygen species [33, 34]. On the other hand, it should be noted that although attenuated, the association between CGI and mortality remained largely significant after further adjustment for HbA1c, mean glucose during hospitalization and CV. This implies that CGI may provide added value in mortality prediction among critically ill patients beyond these conventional glycemic metrics. Additionally, as patients with higher CGI had presumably healthier glucose regulatory system, they may have better glucose control after ICU discharge, which may partially explain the higher long-term survival rate in these subjects.

It is interesting that lower CGI (instead of higher CGI) was observed to be related to worsen glucose control and poorer outcomes. Lundelin et al. [31] hypothesized that a healthy glucose regulatory system should be able to detect small changes in glucose concentrations and makes continuous small adjustments, to maintain glucose homeostasis. Thus, for patients with normal physiological conditions, the tracing of glucose concentration would be characterized by frequent small ups and downs, displaying high glucose complexity. In contrast, in disease status, the glucose regulatory system may require bigger changes in glucose concentration to launch a counterregulatory response, displaying low glucose complexity.

Beyond critically ill patients, existing evidence showed that decreasing CGI is correlated with deteriorating glucose regulation [15]. Moreover, CGI may help identify the residual risk of mortality in people with seemingly well-controlled diabetes [16]. Recently, CGI was found to have a more pronounced association with cognitive dysfunction in type 2 diabetes, compared to HbA1c, SD and time in range [35]. Therefore, CGI holds the promise of complementing the current glycemic assessment system evaluated by CGM as an early and sensitive metric. However, further researches are still warranted to explore the possible role of CGI in diverse populations with abnormal glucose metabolism, and its relationships with different outcomes.

The main strengths of this study include a multi-center, prospective study design, use of 14-day CGM in accordance with recommendation from international consensus and guideline [22], and long-term follow-up. However, there are several limitations that should be noted. First, the diabetes status adjusted in the models may not have been completely accurate. Although we prospectively determined diabetes status at the onset of ICU admission based on all available information, the possibility of undiagnosed diabetes influencing our findings remains. Second, because of a limited scope of the medical records used in our study, the nutrition supply data was not available in this study. Finally, the data on potential risk factors of CGI including mood fluctuations, physical activity, and other environmental factors was not available in the current study. Therefore, the possibility of residual confounding could not be completely excluded.

In conclusion, the present study found that lower CGI is significantly and independently associated with increased short- and long-term mortality in critically ill patients. CGI quantifies the complex non-linear features of glucose regulation, which offers an innovative mean of understanding the systemic properties. Therefore, CGI holds promise to serve as a new marker for assessing glucose homeostasis and predicting poor prognosis in critically ill patients. Its clinical value deserves to be further investigated in different populations.

## Supplementary Information

Below is the link to the electronic supplementary material.

Supplementary file1 (DOCX 7747 KB)

## Data Availability

Restrictions apply to the availability of data generated or analyzed during this study to preserve patient confidentiality or because they were used under license. Data are however available from the authors upon reasonable request.

## Code availability

The underlying code for this study [and training/validation datasets] is not publicly available but may be made available to qualified researchers on reasonable request from the corresponding author.
