# Supplementary material for: The complexity of glucose time series is associated with short- and long-term mortality in critically ill adults: a multi-center, prospective, observational study
Source: J Endocrinol Invest. 2024 May 18;47(12):3091–9. doi: 10.1007/s40618-024-02393-4 (PMC11549136; doi:10.1007/s40618-024-02393-4)
Supplement: Supplementary file 1 — Supplementary file1 (DOCX 7747 KB) [file 40618_2024_2393_MOESM1_ESM.docx]

**Details about the CGI calculation method:**

1. At first, the original CGM time-series data is divided into several non-overlapping segments, and the data points in each segment are averaged to obtain the coarse-grained time series at the corresponding scale. In the present study, time scales were set at 1 to 4. Given that the CGM system recorded a glucose value every 15 min, time scales of 1 to 4 correspond to a period of 15 to 60 min.

2. Subsequently, SampEn is computed on each coarse-grained time series using the RCMSE method (the details of RCMSE could be seen in [1,2]) to quantify the complexity of the CGM time series data at that scale. The following formula were used here:

$$\mathrm{RCMSE}\left( x,\tau,m,r \right)=-\ln\frac{\sum_{k=1}^{\tau} n_{k,\tau}^{m+1}}{\sum_{k=1}^{\tau} n_{k,\tau}^{m}}$$

$$n_{k,\tau}^{m}=\frac{num(d_{ij}<r)}{N-m-1}$$

Where $m$is the embedding dimension, here the embedding dimension is taken to be 2; $r$is the threshold of similarity, which is 20% of the standard deviation of the data series; $k$ is the number of time scales; $n_{k,\tau}^{m}$ and $n_{k,\tau}^{m+1}$ are the number of matched vector pairs at each scale factor τ; $N$is the total number of data points in the coarsely grained time series at each scale factor τ; $d_{ij}$ means the maximum absolute value of the difference between corresponding elements.

3. Finally, CGI was defined as the sum of the four entropy values generated in the previous step, which was calculated as follows:

$$CGI=\sum_{\tau=1}^{4} \mathrm{RCMSE}\left( x,\tau,m,r \right)$$

And we also averaged the CGI values obtained for each day, resulting in one mean value per patient for further analyze.

Reference:

1. Wu S D, Wu C W, Lin S G, et al. Analysis of complex time series using refined composite multiscale entropy[J]. Physics Letters A, 2014, 378(20): 1369-1374.

2. Richman J S, Lake D E, Moorman J R. Sample entropy[M]. Methods in enzymology. Academic Press, 2004, 384: 172-184.

**Supplementary Figure 1. Flow chart of the study.**

**
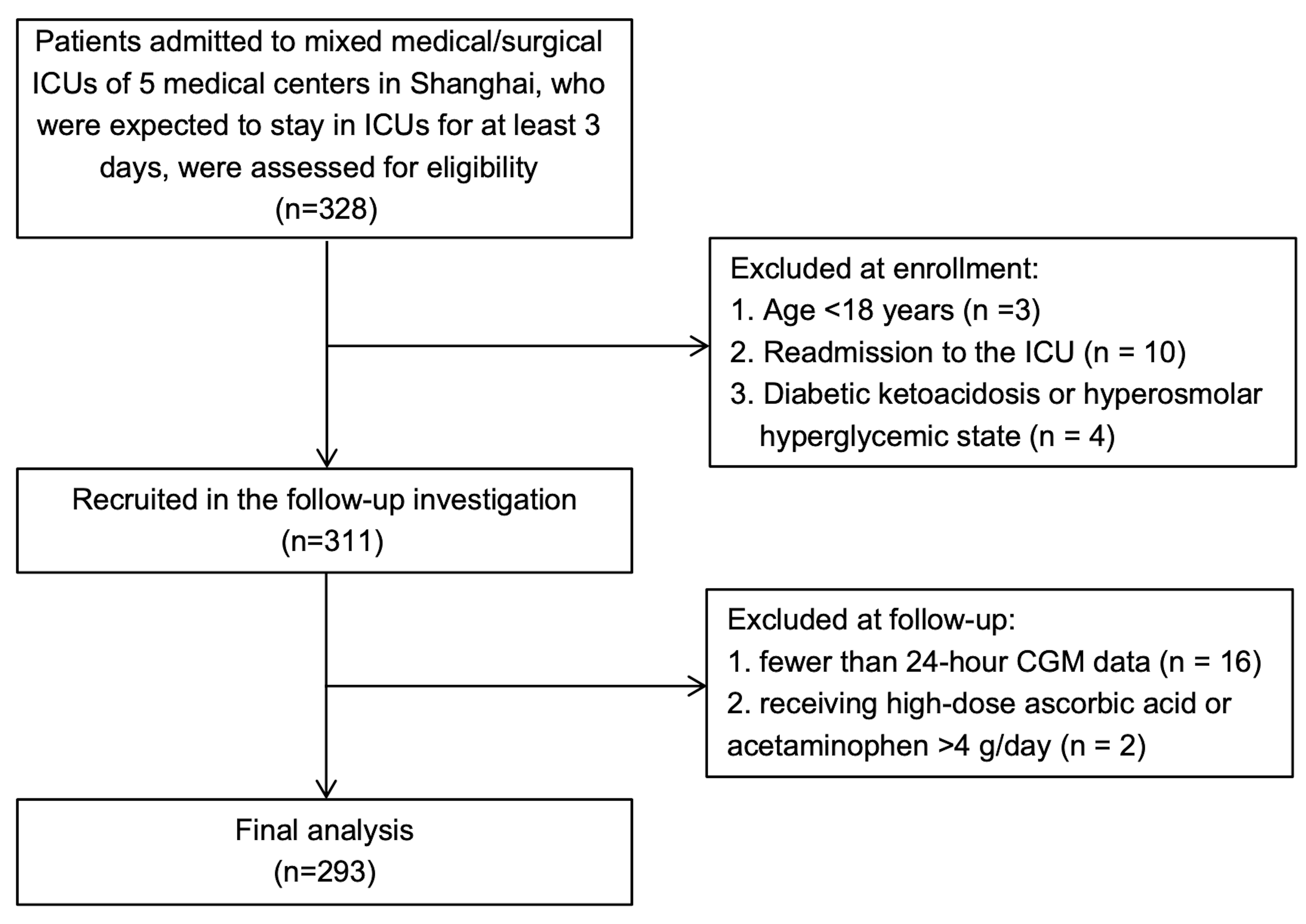
**

**Supplementary Figure 2**. **The HRs of CGI for short- and long-term mortality in critically ill patients using restricted cubic spline analysis.** HRs were adjusted for sex, age, APACHE II score, mechanical ventilation, creatinine, diabetes, use of glucocorticoid in hospital, use of insulin in the hospital, HbA1c, mean glucose during hospitalization and CV. Abbreviation: HR, hazard ratios; CGI, complexity of glucose time series index; HbA1c, glycated hemoglobin A1c; CV, glucose coefficient of variation.


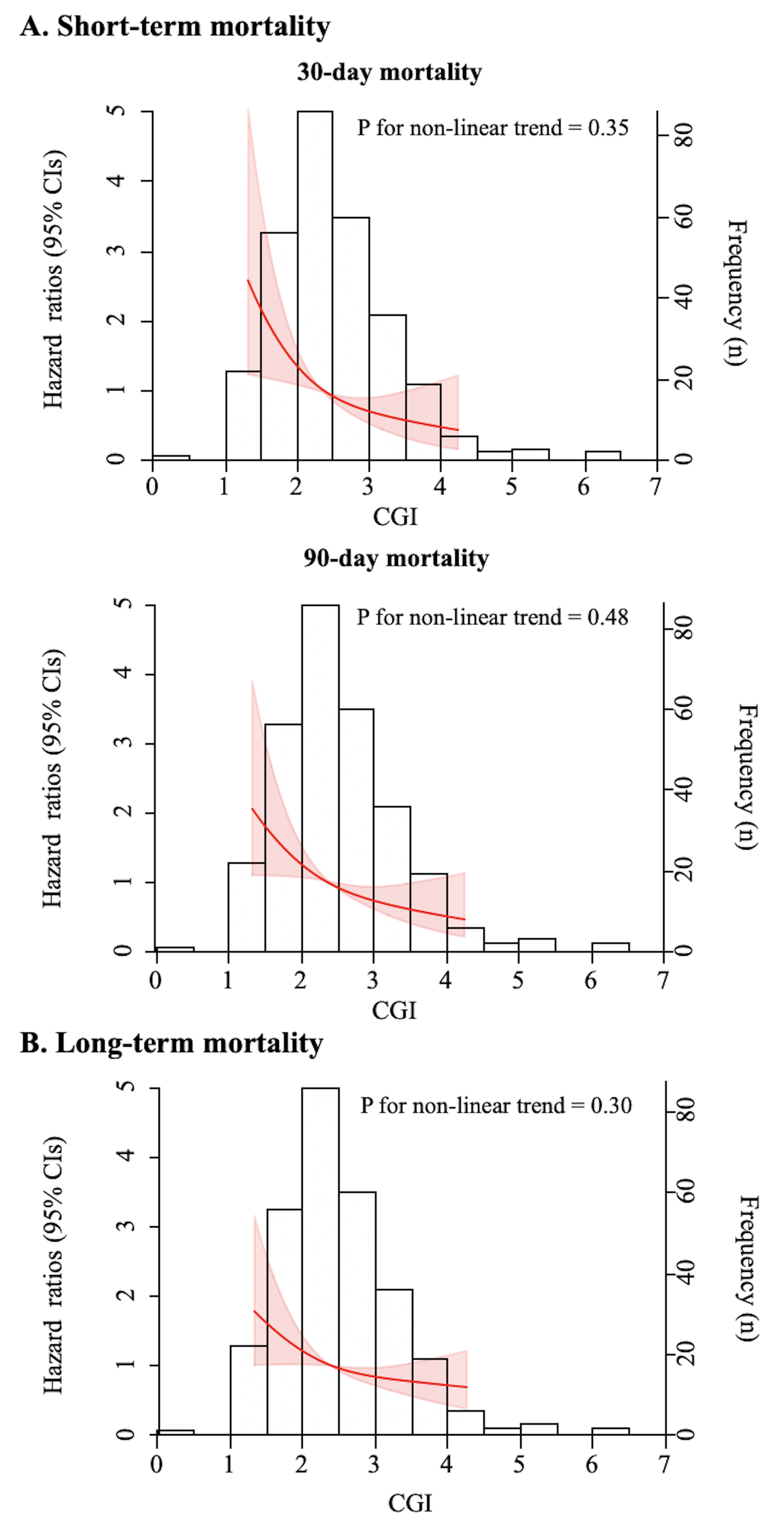


**Supplementary table 1. Spearman correlation coefficient analysis of CGI and other glucose metrics.**

| Variables | *r* | *P* values |
| --- | --- | --- |
| Glycated hemoglobin A1c (HbA1c), % | -0.28 | < 0.001 |
| Mean sensor glucose, mmol/L | -0.55 | < 0.001 |
| Coefficient of variation (CV), % | -0.34 | < 0.001 |
| Standard deviation (SD), mmol/L | -0.60 | < 0.001 |
| Time above range (TAR) > 180 mg/dl (10 mmol/L), % | -0.59 | < 0.001 |
| Time in range (TIR) 70-180 mg/dl (3.9-10 mmol/L), % | 0.42 | < 0.001 |
| Time below range (TBR) < 70 mg/dl (3.9 mmol/L), % | -0.37 | < 0.001 |

**Supplementary table 2. Comparison of the predictive performance for mortality among CGI and other glycemic metrics.**

|  | Basic model  +CGI | Basic model  +HbA1c | Basic model  +mean glucose | Basic model  +CV | Basic model  +TIR |
| --- | --- | --- | --- | --- | --- |
| Short-term mortality |  |  |  |  |  |
| 30-day mortality |  |  |  |  |  |
| C-statistic (95% CI) | 0.74 (0.68-0.80) | 0.69 (0.63-0.76) | 0.71 (0.65-0.78) | 0.71 (0.64-0.78) | 0.71 (0.64-0.78) |
| P for change in C-statistic | Ref. | 0.94 | 0.87 | 0.86 | 0.91 |
| 90-day mortality |  |  |  |  |  |
| C-statistic (95% CI) | 0.75 (0.69-0.81) | 0.72 (0.65-0.78) | 0.73 (0.67-0.79) | 0.72 (0.66-0.79) | 0.74 (0.68-0.80) |
| P for change in C-statistic | Ref. | 0.94 | 0.80 | 0.91 | 0.75 |
| Long-term mortality |  |  |  |  |  |
| C-statistic (95% CI) | 0.74 (0.68-0.80) | 0.74 (0.68-0.79) | 0.74 (0.68-0.80) | 0.73 (0.68-0.79) | 0.74 (0.68-0.80) |
| P for change in C-statistic | Ref. | 0.57 | 0.44 | 0.64 | 0.48 |

Adjusted for sex, age, APACHE II score, mechanical ventilation, creatinine, diabetes, use of glucocorticoid in hospital, and use of insulin in the hospital. CGI, HbA1c, mean glucose, CV and TIR (3.9-10 mmol/L) were analyzed as continuous variables (per 1-SD increase/decrease).

Abbreviation: CGI, complexity of glucose time series index; HbA1c, glycated hemoglobin A1c; CV, glucose coefficient of variation; TIR, time in range.
